# Meat-Inspection

**Published:** 1900-04

**Authors:** 


### COMMUNICATION.

### MEAT-INSPECTION.

EDITOR OF THE JOURNAL OF COMPARATIVE MEDICINE:

I HAVE read with considerable consternation of the proposed legislation in Germany respecting the importation of meat from this country, and could hardly account for the stand taken. I have, however, just been reading the paper on "Municipal Meat-inspection," as reported in the proceedings held some time back in New York, and therein, to my mind, is contained plenty of grounds for forming a poor opinion of our system of inspection, and thus affording an opportunity to take a stand against our meat products by those interested in so doing. The paper gives considerable evidence as to establishing the fact that our system is a long way from being what it should be, and when these facts are published so that they can be had by most anybody, particularly those who are interested in finding fault, is it any wonder that other countries should take a stand in the interest of their own country against our meat, and thus criticise our system of inspection. I trust the Association will take hold of this matter and bring about, in some way, a better condition of affairs, as it is one of the most important subjects touching our profession in relation to its public health service.

I feel the JOURNAL can help notoriety in the matter by setting a foundation of ideas of what is needed, and how it can be brought about. This paper openly acknowledges a condition of affairs that certainly is not pleasant for home consumers, and when we are not over-particular about what our own people eat, why, it is naturally to be presumed we do not care as much about other people as we do our own, or, on the other hand, if we are careless about our home consumption, are they not at liberty to presume we are about others? Mr. Editor, it should be your duty to see that this matter does not rest at the publication of self-criticism. NON LIBET.

